# Administration of rAAV Encoding Monoclonal Antibodies Protects Mice Challenged with a Lethal Dose of H3N2 Influenza Virus and Neutralizes H1 and H3 Strains

**DOI:** 10.32607/actanaturae.27774

**Published:** 2026

**Authors:** R. R. Mintaev, F. A. Urusov, A. V. Soloviev, B. V. Belugin, D. V. Glazkova, G. A. Shipulin, E. V. Bogoslovskaya

**Affiliations:** Federal State Budgetary Institution “Center for Strategic Planning and Management of Medical and Biological Health Risks”, Federal Medical-Biological Agency, Moscow, 119121 Russia

**Keywords:** rAAV, monoclonal antibodies, CR9114, MHAA4549A, MEDI8852, C585, 1G01, influenza

## Abstract

The influenza virus causes seasonal epidemics throughout the world. At the same time, the rapid mutation of the virus renders the use of seasonal vaccines less effective. One of the approaches sought to improve influenza prevention is the use of monoclonal antibodies that are active against a wide range of influenza virus strains. In this study, the virus-neutralizing activity of the monoclonal antibodies CR9114, MHAA4549A, MEDI8852, C585, and 1G01 against the influenza virus was assessed. To this end, recombinant vectors based on adeno-associated virus (rAAV) encoding these antibodies were used. The rAAV vectors were expressed in mice *in vivo*, and the virus-neutralizing activity of the sera against the H1N1 and H3N2 influenza virus strains was assessed. Administration of rAAV-C585, rAAV-MHAA4549A, and rAAV-1G01 conferred 100% protection to mice challenged with a lethal dose of the H3N2 influenza virus. The efficacy of rAAV-CR9114 and rAAV-MEDI8852 against this influenza virus strain was lower, at 80 and 75%, respectively.

## INTRODUCTION

Influenza remains a major healthcare problem worldwide. A total of 3,000,000–5,000,000 severe infection cases and up to 650,000 deaths occur in the world annually, with up to 100,000 of these cases being children under five years of age [1]. Influenza viruses are characterized by a rapid evolution, which reduces the effectiveness of seasonal vaccines [2] and highlights the urgent need to develop novel approaches to influenza treatment and prevention.

The use of monoclonal antibodies active against a broad range of influenza strains represents a promising approach. The antiviral effect of antibodies is mediated by their ability to neutralize viral particles, as well as by effector functions such as antibodydependent cellular cytotoxicity (ADCC) and complement- dependent cytotoxicity (CDC) [3, 4]. Passive immunization with monoclonal antibodies is considered a promising strategy against infectious diseases [5, 6, 7]. The use of monoclonal antibodies is particularly relevant in cases requiring the infection to be rapidly controlled to prevent the development of complications. Passive immunization with monoclonal antibodies has demonstrated effectiveness in severe COVID-19 [8, 9]. At the same time, clinical studies on broadly neutralizing anti-influenza antibodies have demonstrated either no significant differences between experimental and control groups or only minor differences [10, 11, 12].

Monoclonal anti-influenza antibodies may be effective in disease prevention, if their body levels are maintained over a sufficiently long period of time. Long-term presence of antibodies of interest can be achieved by delivery using recombinant adeno-associated virus (rAAV) vectors carrying antibody-encoding genes. To date, rAAV vectors encoding monoclonal antibodies against Ebola [13, 14], HIV-1 [15, 16], and influenza viruses have been developed.

A large number of anti-influenza antibodies have been described [5], which allows for the development of antibody-encoding rAAV and selection of the most promising candidates. Based on the published data on their breadth of activity and efficacy, we selected four antibodies targeting hemagglutinin (HA), namely CR9114, MHAA4549A, MEDI8852, and C585; and one antibody targeting neuraminidase (NA), 1G01 [19, 20, 21, 22, 23].

The antibodies CR9114, MHAA4549A, and MEDI8852 target the HA-conserved stem region. According to [19], CR9114 binds HA of both group 1 and 2 influenza viruses, although it shows a weaker neutralizing activity against the latter. Unlike CR9114, the antibodies MHAA4549A and MEDI8852 exhibit broader activity across both viral groups [20, 24].

Antibodies targeting the HA head region typically neutralize influenza viruses effectively. However, their breadth of activity is limited due to the high variability of the region. Nevertheless, Qiu et al. identified the antibody C585, which targets the HA head region and neutralizes a broad range of H3 viruses [22]. Although C585 is active only against the H3 subtype, it remains of interest because it both binds HA with high affinity and neutralizes numerous H3N2 strains that circulated between 1968 and 2016 [22].

The final antibody selected, 1G01, targets influenza NA. Stadlbauer et al. demonstrated that this antibody protects against a broad spectrum of influenza A and B viruses and exhibits activity against all subtypes (N1–N9, NB) [23].

The aim of the present study is to evaluate the feasibility of using serotype 9 rAAV vectors encoding the monoclonal antibodies CR9114, MHAA4549A, MEDI8852, C585, and 1G01 for protection against influenza in a lethal mouse model.

## EXPERIMENTAL

**Cloning of plasmid vectors for rAAV production**

Amino acid sequences of the antibodies CR9114, MHAA4549A, MEDI8852, C585, 1G01, and COV2-2196 (as a negative control) were back-translated to nucleotide sequences, and the resulting codons were optimized for expression in human cells (*Supplementary Table S1*) using the SnapGene software (GSL Biotech LLC, http://www.snapgene.com/products/snapgene/). In order to reduce the potential immunogenicity of transgenes due to activation of the Toll-like receptor 9 by CpG motifs in foreign DNA, CpG-containing codons were replaced with synonymous ones. The resulting nucleotide sequences were cloned into a plasmid vector whose structure is described in the study by Shipulin et al. [25].

**rAAV production**

Serotype 9 rAAV (rAAV9) was produced in HEK293FT cells (Invitrogen, USA) by co-transfection of the vector plasmid with the plasmids pAAV-Helper and pAAV-RC9 (Cell Biolabs, USA). Vector particles were purified by iodixanol gradient ultracentrifugation. The rAAV9 titer was determined by digital droplet PCR. A detailed protocol for rAAV production, purification, and quantification has been previously described by Shipulin et al. [25].

**Enzyme-linked immunosorbent assay (ELISA)**

Human serum IgG concentrations were measured using the Human IgG ELISA Antibody Pair Kit (STEMCELL, Canada) according to the manufacturer’s instructions. All serum samples were pre-inactivated at 56°C for 30 min.

**Influenza viruses**

To assess the antiviral activity of antibody-encoding vectors, the influenza strains A/California/04/2009 (H1N1) and A/Aichi/2/1968 (H3N2) were used. The strains were provided by the Federal State Budgetary Scientific Institution “Federal Research Center for Fundamental and Translational Medicine.” MDCK. STAT1 KO cells (CCL-34-VHG, ATCC, USA) were used for virus production, titration, and assessment of antibody neutralizing activity. Cells were maintained in complete DMEM (Gibco, USA) supplemented with 10% fetal bovine serum (FBS; Gibco) with incubation at 37°C in a 5% CO_2_ atmosphere. For virus production, the cells were cultured to 90% confluence, after which the medium was replaced with DMEM containing 0.0002% trypsin (“PanEco”, Russia), and viral suspension was added at a dose of 100 × TCID50. The cells were incubated for four days until complete loss of monolayer confluence. The culture medium was then filtered through 0.45-μm filters (TPP, Switzerland) and concentrated 100-fold using Amicon Ultra-15 centrifugal concentrators with a 100 kDa cutoff (Millipore, Germany). Virus concentrate aliquots were stored at –80°C.

TCID50 of influenza viruses were determined using the standard approach (Supplementary protocol 4) previously described in [26]. Virus titers were assessed visually based on the loss of cell monolayer confluence (cytopathic effect; CPE). Titers were calculated using the Reed and Muench method [27].

**Experimental animals**

Female C57BL/6 mice aged 6 weeks and weighing 16–18 g were used in the study. The animals were provided by the Stolbovaya nursery, a branch of the Federal State Budgetary Scientific Institution “Scientific Center for Biomedical Technologies of the Federal Medical and Biological Agency”.

**Adaptation of the A/Aichi/2/1968 (H3N2) virus and LD_50_ assessment**

**(H3N2) virus and LD_50_ assessment** The A/Aichi/2/1968 (H3N2) virus was adapted to C57BL/6 mice using the previously described method (Basic protocol 2) [26]. To evaluate the virus LD_50_, five groups of six animals each were formed. The groups were infected intranasally with the virus at 10^0^, 10^-1^, 10^-2^, 10^-3^, and 10^-5^ dilutions (Supplementary protocol 5) [26]. The number of survived animals was assessed in each group after 17 days, and LD_50_ was calculated using the Reed and Muench method [27].

**Passive immunization and evaluation of the protective activity of rAAV constructs**

To study the protective activity of rAAV constructs* in vivo*, six experimental groups of ten mice each were formed. All the groups received intramuscular injections of the corresponding rAAV at a dose of 2 × 10^11^ vector genomes (vg) per mouse in 100 μL. Five groups received rAAV encoding anti-influenza antibodies (groups 1–5), while the sixth group received rAAV encoding the COV2-2196 antibody against SARS-CoV-2 and served as a negative control. An additional control group comprised of 22 animals did not receive any rAAV. After 90 days of rAAV administration, the animals were challenged intranasally with 30 μL of the adapted A/Aichi/2/1968 (H3N2) virus at a dose of 5 LD_50_. Animal survival and body weight were monitored on a daily basis for 14 days in all groups.

**Influenza virus neutralization**

A virus neutralization assay was performed according to the previously described protocol (Supplementary protocol 11) [26]. Serial two-fold dilutions (1:10–1:160) of serum samples were prepared in complete DMEM in 96-well plates. The resulting dilutions were mixed with the influenza virus at a dose of either 25 or 8 TCID50 in a final volume of 100 μL. After 1 h of incubation, 100 μL of MDCK.STAT1 KO cells were added to each well (2.5 × 10^4^ cells per well). The complete culture medium was replaced with DMEM supplemented with 0.0002% trypsin after 24 h. Viral CPE was assessed after 72 h. The IC_50_ values were calculated using the Reed and Muench method [27].

**Statistical data analysis**

Differences in survival rates between experimental groups were assessed using the Mantel–Cox test (*p*-value < 0.05). Differences in IgG antibody titers in mouse serum were evaluated using the Kruskal– Wallis test, followed by Dunn’s multiple comparison test with Bonferroni correction for multiple comparisons. A *p*-value < 0.01 was considered statistically significant.

## RESULTS

**Assessment of antibody expression *in vivo***

**Fig. 1:**
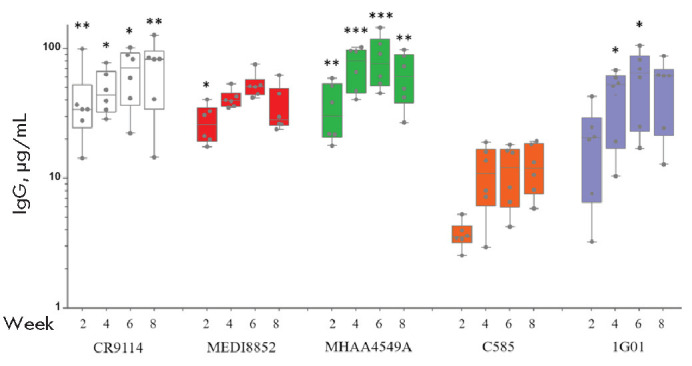
Human IgG levels in mouse serum. Individual values are presented for each animal (*n *= 6). Horizontal lines in boxes represent the median. The box boundaries indicate quartiles Q1 (25%) and Q3 (75%). Whisker tips denote the minimum and maximum values. Differences in IgG antibody titers in mouse serum were analyzed using the Kruskal–Wallis test followed by Dunn’s multiple comparison test with Bonferroni correction. Statistically significant differences between the C585 antibody titer and the titers of other antibodies at the same time points are indicated by asterisks: *p-value < 0.01, **p-value < 0.001, ***p-value < 0.0001

We used the plasmid vectors rAAV-CR9114, rAAV-MHAA4549A, rAAV-MEDI8852, rAAV-C585, and rAAV-1G01 to express antibodies in the IgG1 format under the control of the hybrid CMV/EF1 alpha promoter. The plasmids were used for rAAV production in HEK293FT cells, followed by vector administration to C57BL/6 mice at a dose of 2 × 10^11^ vg per mouse. At the first stage, the ability of rAAV vectors to mediate antibody expression *in vivo *was assessed. Serum levels of human antibodies were measured in mice at weeks 2, 4, 6, and 8 after rAAV administration (*Fig. 1*). Antibody levels increased over time and reached their maximum at week 6. At week 8, median levels of the antibodies CR9114, MEDI8852, MHAA4549A, and 1G01 were 82, 27, 60, and 61 μg/mL, VOL. 18 № 1 (68) 2026 | ACTA NATURAE | **67** respectively. In contrast, the median C585 level was 12 μg/mL, which is significantly lower than those of the other antibodies.

**Determination of the antiviral activity of sera from immunized mice by virus neutralization assay**

After the primary evaluation of rAAV-mediated antibody expression *in vivo*, a second experiment was conducted in larger animal groups (8–10 C57BL/6 mice per group). Each animal received a single injection of the corresponding rAAV at a dose of 2 × 10^11^ vg per mouse. Control animals did not receive rAAV. Mice injected with rAAV encoding the SARSCoV- 2-specific antibody COV2-2196 were used as an additional negative control [28].

**Table 1:** Neutralization of A/Aichi/2/1968 (H3N2) and A/California/04/2009 (H1N1) with mouse serum pools (virus dose, 25 TCID50)

Serum pool/ animal group	Vector	IgG concentration, μg/mL	A/California/04/2009 (H1N1)		A/Aichi/2/1968 (H3N2)	
			Dilution	IC_50_, μg/mL	Dilution	IC_50_, μg/mL
1	rAAV-CR9114	83.7	1 : 10	3.090	–	–
2	rAAV-MEDI8852	39	1 : 20	1.379	1 : 10	1.379
3	rAAV-MHAA4549A	57.8	1 : 20	1.445	1 : 20	1.022
4	rAAV-C585	12.5	–	–	1 : 40	0.110
5	rAAV-1G01	55.5	1 : 20	1.189	1 : 20	1.025
6	rAAV-2196	24.5	–	–	–	–

Note. The maximum serum dilution providing protection and IC_50_ values are indicated. The “–” denotes absence of neutralization.

Four weeks after rAAV administration, serum samples from each experimental group were pooled in equal volumes. Antibody levels in the resulting serum pools were comparable to those observed in the first experiment (*Table 1*). The serum pools were studied in a neutralization assay against the A/Aichi/2/1968 (H3N2) and A/California/04/2009 (H1N1) viruses. Neutralizing dilutions of the serum pools and the corresponding IC_50_ values are presented in *Table 1*.

Theantibodies CR9114, MEDI8852 , and MHAA4549A, which target the HA stem region, neutralized A/California/04/2009 (H1N1) with IC_50_ values of 3.090, 1.379, and 1.445, respectively. Among the antibodies studied, the NA-targeting antibody 1G01 demonstrated the highest neutralizing activity against A/California/04/2009 (IC_50_ = 1.189). The C585 antibody, which is specific to the HA of H3 subtype viruses, did not exert any neutralizing activity against H1 subtype viruses, as expected.

**Table 2:** Neutralization of A/Aichi/2/1968 (H3N2) and A/California/04/2009 (H1N1) with mouse serum pools (virus dose, 8 TCID50)

Serum pool/ animal group	Vector	IgG concentration, μg/mL	A/California/04/2009 (H1N1)		A/Aichi/2/1968 (H3N2)	
			Dilution	IC_50_, μg/mL	Dilution	IC_50_, μg/mL
1	rAAV-CR9114	83.7	1 : 40	0.892	1 : 10	3.566
4	rAAV-C585	12.5	–	–	1 : 160	0.029
6	rAAV-2196	24.5	-	-	-	-

Note. The maximum serum dilution providing protection and IC_50_ values are indicated. The “–” denotes absence of neutralization.

C585 exhibited the highest effectiveness against A/Aichi/2/1968 (H3N2), with an IC_50_ of 0.110. The antibodies MEDI8852, MHAA4549A, and 1G01 showed similar, although one order of magnitude lower, neutralizing activity against the H3 virus. Serum samples from the animals receiving CR9114 did not neutralize A/Aichi/2/1968. We hypothesized that the serum level of CR9114 was insufficient to achieve virus neutralization at the viral dose used (25 TCID50). For this reason, neutralization with CR9114, C585, and the control antibody COV2-2196 was conducted using a virus dose of 8 TCID50. Under these conditions, CR9114 did not exhibit any neutralizing activity against A/Aichi/2/1968 even at low serum dilutions (*Table 2*).

Thus, rAAV administration to mice ensures *in vivo* production of antibodies with distinct neutralization profiles. Next, we assessed the protective efficacy of each AAV vector against A/Aichi/2/1968 (H3N2) in a lethal mouse model.

**Assessment of the protective efficacy of antibodies against A/Aichi/2/1968 in a lethal mouse model**

**Fig. 2:**
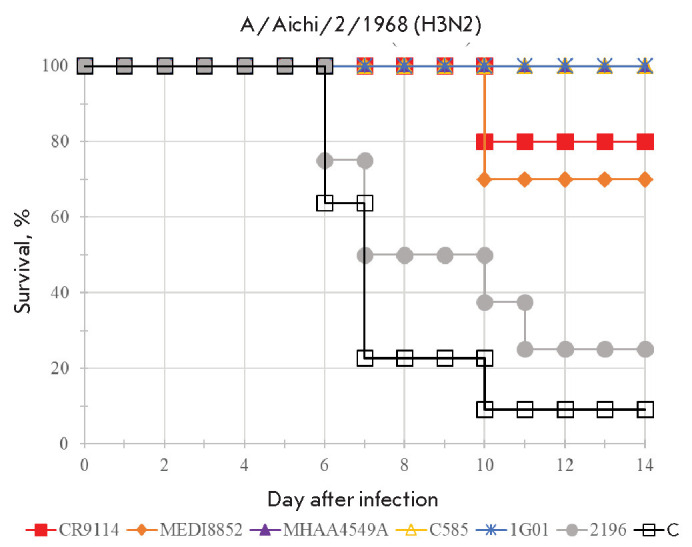
Survival of mice passively immunized with rAAV in a mouse model of influenza-induced pneumonia. The animals were challenged with A/Aichi/2/1968 (H3N2). A statistically significant difference (*p* < 0.05; Mantel–Cox text) was observed between the control and each group receiving rAAV encoding anti-influenza antibodies. C – negative control

The protective efficacy of the antibodies was assessed 3 months after rAAV administration. The mortality rate in the control animals receiving rAAV-COV2-2196 was first recorded on day 6 after infection with A/Aichi/2/1968 (H3N2). By day 11, mortality had reached 90.8% in the control group and 75% in the COV2-2196 group (*Fig. 2*, *Supplementary Table S2*).

**Fig. 3:**
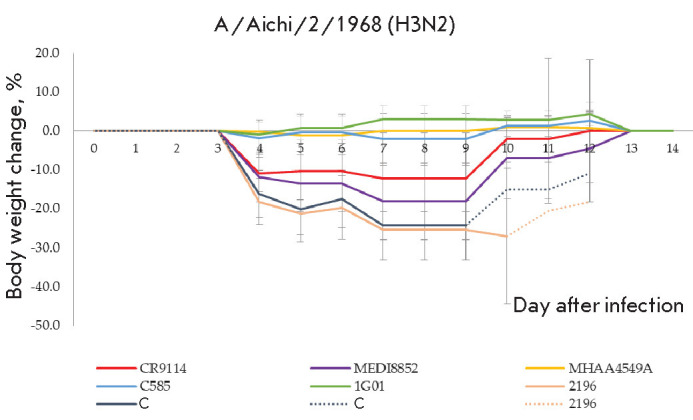
Changes in the body weight of mice challenged with A/Aichi/2/1968 (H3N2) following passive immunization with rAAV. Mean values ± SD are shown. Dashed lines indicate body weight changes in the corresponding groups without SD (the number of mice per group is < 3). C – negative control

A statistically significant (*p*-value < 0.05) increase in animal survival results was noted in all groups undergoing passive immunization with rAAV vectors encoding anti-influenza antibodies. No deaths were recorded in mice immunized with rAAV-MHAA4549A, rAAV-C585, and rAAV-1G01, although an insignificant decrease in the body weight was noted at several time points. At the same time, 20 and 30% of the animals receiving the antibodies CR9114 and MEDI8852, respectively, died. The body weight in these groups decreased by 15 and 20%, respectively, which was an indication of incomplete protection (*Fig. 3*).

## DISCUSSION AND CONCLUSIONS

The potential use of broadly neutralizing antibodies for influenza treatment and prevention has been under active investigation in recent years [5, 29]. Since the influenza virus is highly mutable, its prevention requires antibodies that act against the broadest possible range of influenza strains. In this work, we conducted the first simultaneous comparison of the effectiveness of rAAV vectors designed to express five previously characterized anti-influenza antibodies. We evaluated the neutralizing activity of the constructed antibodies against two viral strains, namely A/California/04/2009 (H1N1) and A/Aichi/2/1968 (H3N2), which represent the most distant subgroups (groups 1 and 2) within influenza A viruses [30].

The rAAV-mediated expression of the HA stemspecific antibodies MHAA4549A and MEDI8852 ensured virus-neutralizing activity of mouse serum against both the H1N1 and H3N2 strains, which is consistent with the previously reported results [20, 21]. The rAAV-CR9114 vector provided antibody production in serum sufficient to neutralize only the H1 virus at a virus dose of 25 TCID50 (*Table 1*). Serum samples from mice receiving rAAV-CR9114 neutralized both the H1 and H3 strains at a reduced virus dose of 8 TCID50. The obtained data are consistent with the previously reported findings that CR9114 is significantly more effective against group 1 than group 2 influenza A viruses [31]. Despite the relatively weak neutralizing activity of CR9114-containing serum samples against A/Aichi/2/1968 (H3N2), the rAAV-CR9114 vector protected 80% of lethally infected mice from death. A similar observation was reported by Dreyfus et al. [19], who showed that the CR9114 antibody protected challenged animals from death even despite its weak neutralizing activity against H3N2 and the inability to neutralize influenza B viruses. However, this protection was accompanied by a 10% loss in body weight. In cases of weak neutralization, the protective effect of CR9114 was shown to be mediated by Fc-dependent mechanisms [32].

Determining the relative contributions of the neutralizing activity and Fc-mediated effector functions to the protective effect of antibodies cannot always be done in a straightforward fashion. For instance, serum samples from mice injected with rAAV-MHAA4549A and rAAV-MEDI8852 exhibited a similar neutralizing effect against A/Aichi/2/1968 (H3N2), whereas only rAAV-MHAA4549A conferred 100% protection to mice without a noticeable decrease in body weight. This phenomenon may be due to differences in either antibody expression levels in mice or the efficiency of the Fc-mediated effector functions, namely ADCC and ADCP [21, 33].

Among the antibodies studied, particular attention should be paid to rAAV-1G01, which encodes a neuraminidase (NA)-specific antibody with activity against all influenza virus subtypes (N1–N9, NB) [23]. Furthermore, the 1G01 antibody exhibits neutralizing activity, which distinguishes it from other NA-specific antibodies that primarily inhibit NA enzymatic activity, rather than neutralize the virus. In our experimental study, we confirmed both the neutralizing activity of serum from mice passively immunized with rAAV-1G01 against A/California/04/2009 (H1N1) and A/Aichi/2/1968 (H3N2) and its protective activity against H3N2 infection *in vivo*.

Thus, all the studied vectors demonstrated the ability to statistically significantly protect mice from a H3N2 influenza virus infection and may be used for further development of rAAV-based agents for passive immunization. Further studies are required to evaluate the protective efficacy of these vectors against a broader panel of influenza viruses. If necessary, combinations of individual vectors may be in order to increase the drug breadth and effectiveness. For example, the CR9114 antibody, which primarily targets H1 viruses, could be combined with the H3- specific antibody C585.

The potential side effects associated with the immunogenic properties of rAAV vectors should be also considered [34]. There exist data on negative effects primarily reported during the therapy of hereditary diseases requiring systemic rAAV administration [35]. Intranasal administration of rAAV represents a promising strategy for influenza prevention [17], as it may promote local antibody expression at the primary site of infection. This approach may be a safer alternative to systemic vector administration and could potentially enable the development of an effective long-acting prophylactic agent suitable for immunocompromised patients and other vulnerable groups.

## Abbreviations

ADCC: antibody-dependent cellular cytotoxicity
CDC: complement-dependent cytotoxicity
rAAV: recombinant adeno-associated virus
HA: hemagglutinin
NA: neuraminidase
CPE: cytopathic effect
vg: vector genome
